# Case Report: Diverticulum of the left ventricular outflow tract: multimodal imaging

**DOI:** 10.3389/fcvm.2024.1489688

**Published:** 2024-11-20

**Authors:** Qiqing Chen, Lili Liu, Yiqiu Cao, Pian Chen, Wenjin Zhong, Xiangxiang Jing

**Affiliations:** ^1^ Department of Ultrasound, Hainan General Hospital (Hainan Affiliated Hospital of Hainan Medical University), Haikou, China; ^2^ Department of Cardiology, Hainan General Hospital (Hainan Affiliated Hospital of Hainan Medical University), Haikou, China; ^3^ Department of Pathology, Hainan General Hospital (Hainan Affiliated Hospital of Hainan Medical University), Haikou, China

**Keywords:** congenital heart disease, left ventricular diverticulum, multimodal imaging examination, left ventricular aneurysm, subvalvular aneurysm

## Abstract

The left ventricular diverticulum (LVD) is a rare congenital heart disease commonly discovered during examinations. Herein, we report a LVD on the lateral wall of left ventricular outflow tract. A case of a 22-year-old female patient who visited our hospital with complaints of shortness of breath, accompanied by vomiting, cough, and lower limb and facial edema and was diagnosed with “aortopulmonary septal defect” using transthoracic echocardiography. After cardiac surgery and postoperative pathology, she was confirmed with a LVD. The patient's clinical symptoms, multimodal imaging examination, and treatment methods were used to assess this case.

## Introduction

According to existing literature, the left ventricular diverticulum (LVD) represents an exceedingly uncommon cardiac anomaly with a relatively low prevalence, ranging from 0.02% to 0.76% (1, 2). The majority of LVD cases manifest without overt symptoms and are sporadically identified. An abnormal enlargement of the local ventricular cavity owing to congenital reduction or loss of myocardial tissue in the local wall indicates a congenital LVD. It can be histologically divided into muscular and fibrous types (3). The muscular type is mostly located at the heart apex, with the diverticulum wall synchronously moving with the normal ventricular wall (4). The muscle fibers of fibrous diverticulum are reduced or absent, and the diverticulum wall has no contractile function (5). The fibrous diverticula commonly observed are frequently situated beneath the mitral or aortic valve. Herein, we report a rare case of the left ventricular outflow tract lateral wall diverticulum, pathologically confirmed as a cardiac diverticulum after surgery. Imaging methods such as transthoracic echocardiography (TTE), computed tomography angiography (CTA) and digital subtraction angiography (DSA) were used to evaluate.

## Case report

A 22-year-old female patient reported a history of shortness of breath for several years accompanied by vomiting, cough, and lower limb and facial edema. She had been admitted to our hospital in 2017. During her stay, TTE and CTA were used to diagnose the aortopulmonary septal defect (ASPD) and sinus of Valsalva aneurysm (SVA), respectively. Cardiac catheterization was performed, and establishing the trajectory was unsuccessful. Surgical treatment was recommended. She refused surgery. However, she experienced recurrent shortness of breath and bilateral lower limb edema. She returned to our hospital for medical treatment. Clinical examination revealed that there was a palpable thrill that could be palpated in the precordial area. The boundary of cardiac dullness was slightly enlarged, with a term regular heartbeat. The precordial area and the 2nd and 3rd intercostal space on the left edge of the sternum could be palpated with grade 3/6 systolic murmurs. The patient denied a medical history of hypertension, tuberculosis, etc., and had no history of trauma.

**Figure 1:**
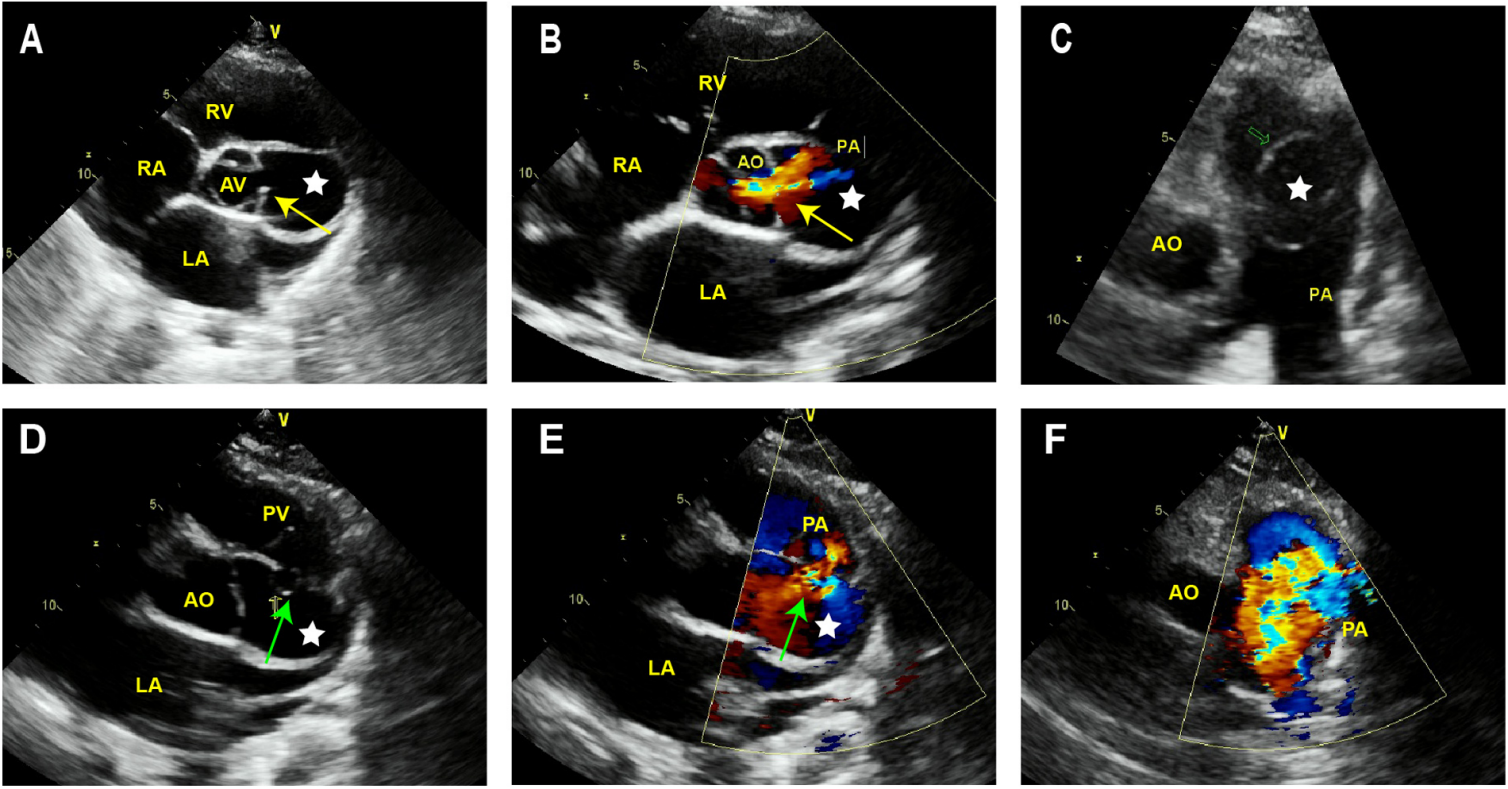
Transthoracic echocardiography of LVD. **(A)** The short axis sections of the aorta depict a cystic structure (white star) between the aorta and pulmonary artery. **(B)** Color Doppler imaging reveals that in the systolic phase, blood exhibits a flow trajectory enters the capsule cavity via the established communication port. **(C)** The long axis section of the pulmonary artery demonstrates that the cystic structure protrudes into the pulmonary artery. **(D,E)** At the top of this cystic structure, a gap of approximately 10 mm width can be observed communicating with the lateral wall of the pulmonary artery. **(F)** Colorful embedded high-speed blood flow signals can be observed in the pulmonary artery. AV, aortic valve; AO, aorta; PA, pulmonary artery; PV, pulmonary valve; LV, left ventricle; LA, left atrium; RV, right ventricle; RA, right atrium.

**Figure 2:**
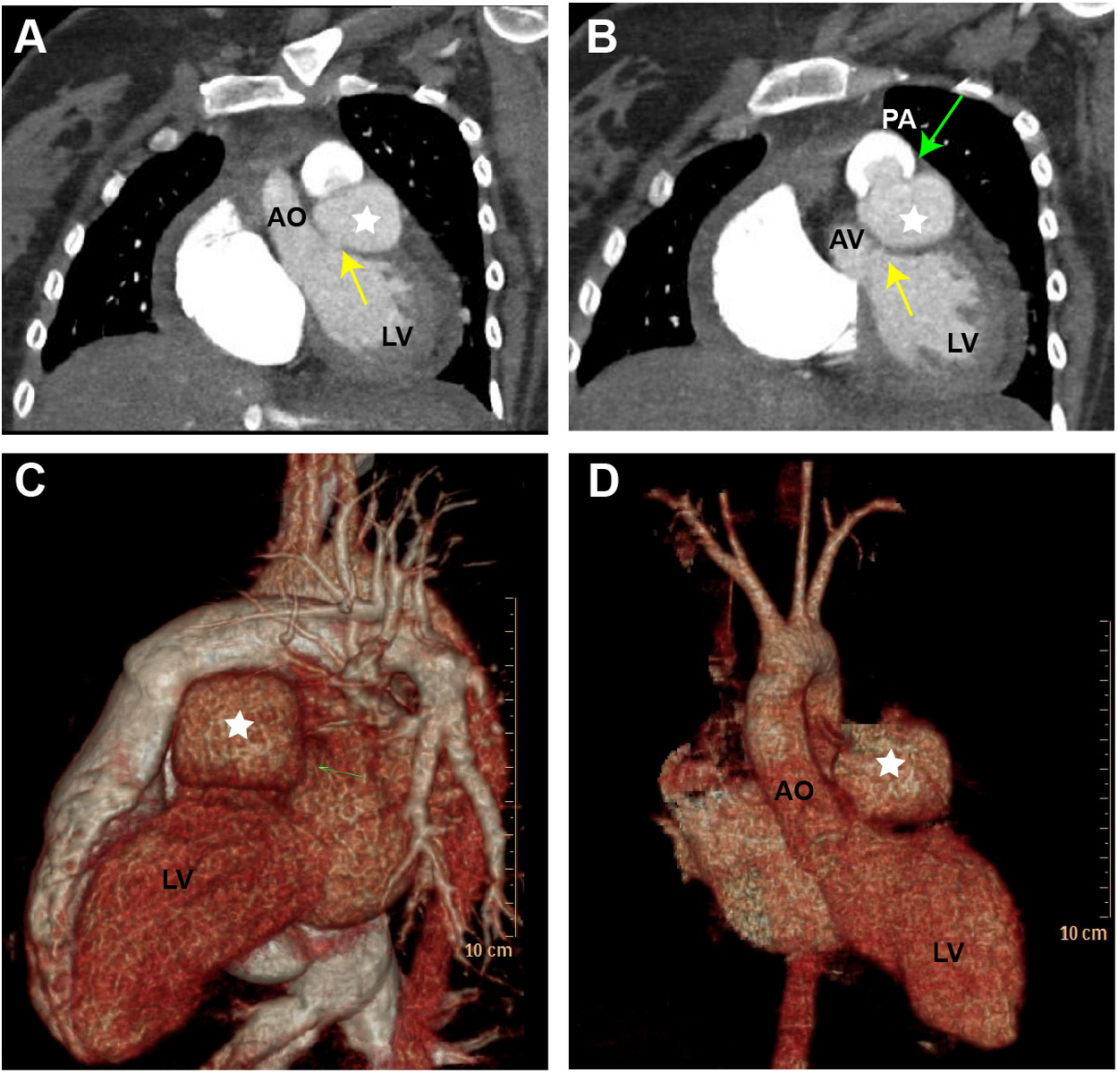
Computed tomography angiography (CTA) and three-dimensional computed tomography (3D-CT) scan of the LVD. **(A)** Display the cystic structure between the aorta and pulmonary artery (yellow arrow). **(B)** The cystic structure protrudes into the pulmonary artery (green arrow). **(C,D)** The 3D-CT scan illustrates the LVD in different views (white star). LV, left ventricle; AO, aorta; AV, aortic valve; PA, pulmonary artery.

**Figure 3:**
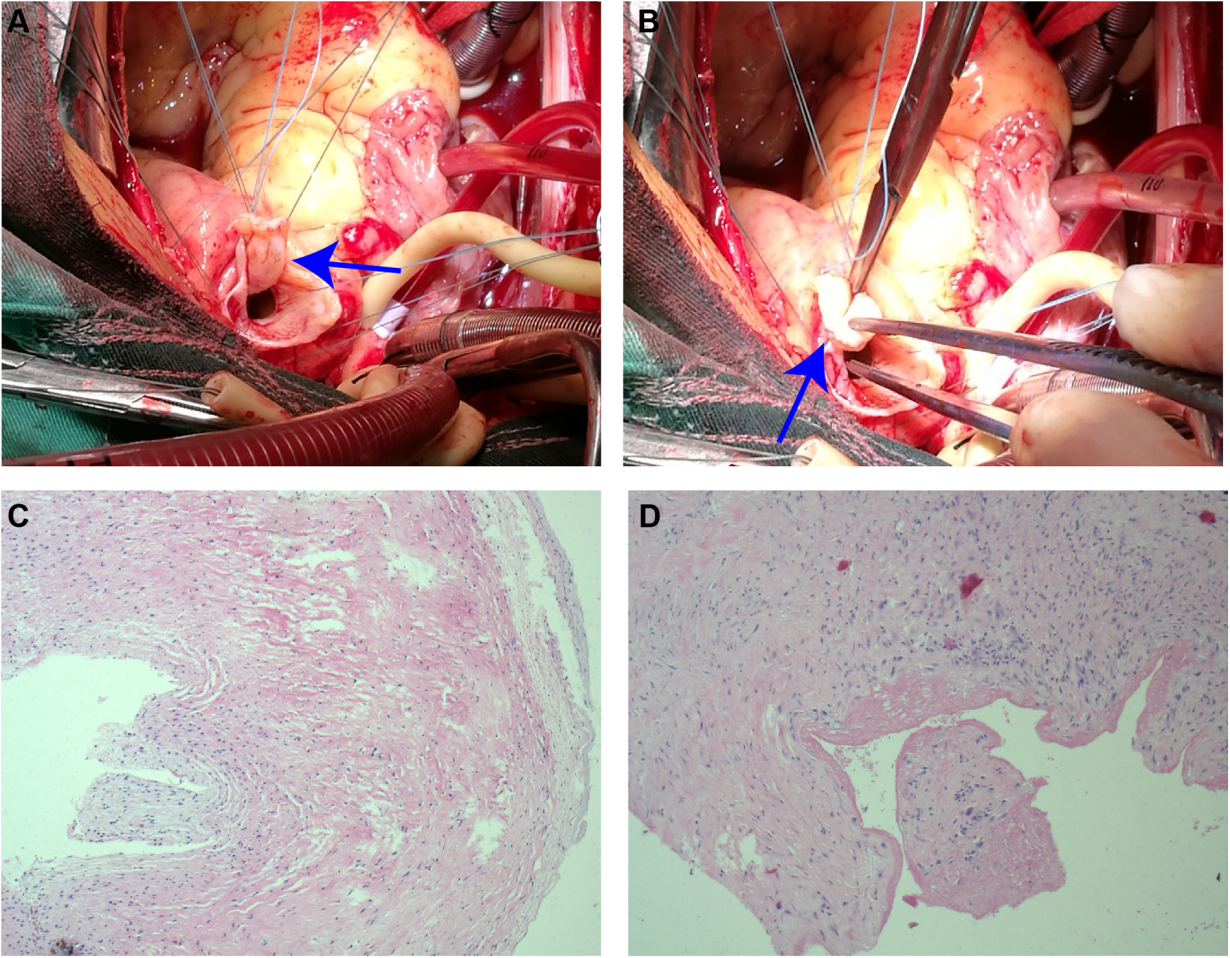
Surgical and pathological results. **(A)** The diverticulum adheres to the lateral wall of the left ventricular outflow tract (closely below the aortic annulus) through the small neck (blue arrow). **(B)** The diverticulum appears as a tumor-like protrusion, extending into the pericardial cavity and pulmonary artery (blue arrow). **(C,D)** Histopathological sections at 10 magnification: no epithelial lining in the capsule wall tissue. Myocardial cell atrophied and fibrosised, with some hyaline degeneration and local mucinous degeneration.

TTE revealed the presence of a left atrium and left ventricular enlargement, a left ventricular ejection fraction (LVEF) of 64%. We misdiagnosed the cystic structure communicating with the aorta through a channel with a width of approximately 7 mm on the side wall of the aortic valve ring (Figures 1A–C); and another communication port with a width of approximately 10 mm was also observed at the top of cystic structure, which connected to pulmonary artery (Figures 1D,E). The morphological changes of the cystic structure were not significant with respect to the contraction of the heart. Color Doppler imaging revealed that blood flowed through the communicating port into the capsule cavity during systole. Subsequently, the blood enters the pulmonary artery. Due to the presence of left to right shunting from the left ventricle to the pulmonary artery, the blood flow velocity in the pulmonary artery trunk increases rapidly, presenting a colorful mosaic-like blood flow signal (Figure 1F). CTA misdiagnosed as a SVA, approximately 4.1 × 3.2 cm in size, the width of the traffic area was approximately 8 mm (Figure 2). The main pulmonary artery was slightly widened, with a diameter of approximately 3.0 cm. Well visualization of the ascending aorta, descending aorta, left pulmonary artery, right pulmonary artery, brachiocephalic trunk, left common carotid artery, and proximal left subclavian artery, with smooth lumens and no stenosis or dilation. Digital subtraction angiography (DSA) revealed no significant stenosis or dilation in the right or left coronary arteries, and DSA suggested the presence of the diverticulum (Supplementary Figure S1). After completing relevant examinations after admission, including laboratory examination results exhibited elevated levels of some biomarkers like leukocyte (11.61 × 10^9^/L; reference value 3.5∼9.5 × 10^9^/L), C-reactive protein level (54.17 mg/L; reference value 0.068∼6.2 mg/L), hypersensitive troponin T (491 μg/L; reference value < 0.014 μg/L), N-terminal forebrain natriuretic peptide BNP (2,741 ng/L; reference value < 300 ng/L). Hepatitis B, hepatitis C, HIV antibodies and syphilis spirochete specific antibodies were negative and the patient's electrocardiogram results showed sinus rhythm. There were no obvious surgical contraindications, and she planed to undergo left ventricular diverticulectomy and pulmonary artery repair surgery. During surgery, a cystic diverticulum could be observed on the lateral wall of the left ventricular outflow tract (Figure 3A), extending toward the pericardial cavity and pulmonary artery. A communication port of approximately 8 mm (located below the aortic annulus) could be observed on the lateral wall of the left ventricular outflow tract and a rupture with a diameter of approximately 1 cm at the top of the capsule (Figure 3B; Supplementary Figure S2), communicating with the pulmonary artery. A continuous suturing technique using a 5-0 Prolene thread was employed to suture the communication port of the left ventricular outflow tract and the port of pulmonary artery rupture. Postoperative pathology revealed no epithelial lining in the capsule wall tissue. Myocardial cell atrophied and fibrosised, with some hyaline degeneration and local mucinous degeneration, consistent with changes in ventricular diverticulum (Figures 3C,D). After the surgery, the patient did not complain of any special discomfort. Her vital signs were stable, and her respiratory sounds were clear through auscultation in both lungs, without any dry or wet rales. There was no obvious murmur in the heart valves, and there was no swelling in the limbs. Postoperative echocardiography demonstrated that the left ventricle was still dilated, and the diverticulum collapsed without any residual shunts (Supplementary Figure S3).

## Discussion

O'Bryan, in 1838 was the first to describe CVD, a rare cardiac anomaly commonly discovered coincidentally in adulthood (1). The diagnosis is based on excluding coronary artery disease, traumatic or inflammatory causes, and other potential cardiomyopathies. Echocardiography is significantly superior to imaging examinations such as CT and magnetic resonance imaging owing to its real-time dynamic observation, and repeatable examination characteristics and has become the preferred method for diagnosing LVD (6, 7). The use of multimodal imaging combined with examination can improve the ventricular diverticulum diagnostic accuracy and play a critical role in selecting treatment methods (3, 8–10).

This article analyzes the causes of misdiagnosis in this case as follows. In prior literature, reports indicated that LVDs were predominantly located at the LV apex. Echocardiographic findings consistently exhibit notable ventricular wall thinning accompanied by localized wall swelling, leading to the formation of a cystic structure. Color Doppler imaging clearly illustrates that the cystic structure maintains a singular connection with the ventricle. Further depiction through Color Doppler imaging demonstrates a bidirectional flow pattern between the ventricle and the cystic cavity during both systole and diastole. However, the case presentation is different from the previously reported manifestations of the ventricular diverticulum. Firstly, the diverticulum location is unique, as it is located below the aortic valve annulus and adjacent to the aorta, making it prone to misdiagnosis as the aortic or aortic sinus lesion. A rupture with a diameter of approximately 1 cm can also be observed at the other end of the diverticulum extending into the pulmonary artery. Color Doppler imaging depicts that systolic blood flows from the left ventricular outflow tract into the capsule through the communicating port. The blood then enters the pulmonary artery through another communicating port at the top of the cystic structure. However, the left ventricular outflow tract diverticulum which ruptured into the pulmonary artery has not been previously reported. This case presentation is quite similar to the location and morphology of the ventricular diverticulum below the aortic valve reported by Zhong et al. (11). However, the ventricular diverticulum reported only has a small orifice below the aortic valve connected to the left ventricular outflow tract, without any rupture at the top of the diverticulum connected to the pulmonary artery. Therefore, owing to an insufficient understanding of congenital diverticulum, the clinical manifestations may not be obvious or specific, and the special manifestations are the primary reasons for misdiagnosis by imaging radiologists.

The differential diagnosis for this case is as follows: A rare congenital cardiovascular malformation, APSD (12, 13). In this case, surgical validation confirmed the diverticulum arising from the lateral wall of the left ventricular outflow tract, situated nearby beneath the aortic annulus. Therefore, the origin of cystic structures and blood flow signals should be determined for differential diagnosis of the diverticulum. In addition, the individual under consideration in this case is a young woman with no prior medical record of conditions associated with SVA, such as Marfan syndrome, trauma, atherosclerosis and iatrogenic injuries during aortic valve replacement (14). Notably, distinct dissimilarities are noted between the location of the lesion and the alterations in hemodynamics. Therefore, this case is not considered as SVA. The third one is left ventricular aneurysm (LVA), which is a serious complication after acute myocardial infarction, often accompanied by pathological Q-wave changes in the ventricular wall and persistent ST-segment elevation (15, 16). LVA is demonstrated on TTE as a thin-walled bag formed in the myocardial infarction area, communicating with the left ventricle through a wide neck. A cystic structure formed by the continuous interruption of the local ventricular wall after myocardial infarction, surrounded by the pericardium or surrounding connective tissue, is referred to as a pseudo ventricular aneurysm. The electrocardiogram of the patient in this case showed sinus rhythm, and DSA showed no significant stenosis in the left and right coronary arteries. Therefore, LVA is not considered. The fourth is subvalvular aneurysm, which is also a key focus of differential diagnosis. Congenital subvalvular aneurysm is a rare anomaly that may be due to congenital weakness between the valve annulus and the myocardial wall that includes submitral and subaortic aneurysm. Subaortic aneurysm usually occurs under the intermediate portion of the left coronary cusp of the aortic valve. The orifice of subaortic aneurysm is usually small which is connected to the aortic root on the ventricular side of the valve. In addition, subvalvular aneurysm has been reported as a sequelae of infective endocarditis (IE) and surgical trauma (17). The patient in this case has no history of IE or surgical trauma. During the surgery, it was confirmed that the cystic structure originated from the left ventricular outflow tract and was connected to the left ventricle rather than the aortic root. Therefore, the medical histories, multimodal imagings, surgical findings and postoperative pathological results are helpful for us to make differential diagnosis.

## Conclusion

In summary, the LVD is rare, without obvious or specific clinical manifestations. The imaging manifestations also show the phenomenon of similar diseases and different maps, making it challenging to distinguish. The LVD can be diagnosed through multimodal examination methods. Among these, echocardiography is the preferred examination method currently, which can evaluate the changes in the morphology, structure, and function of the ventricle and assess the hemodynamic changes of the heart, providing an effective basis for surgery and prognosis evaluation.

## Data Availability

The original contributions presented in the study are included in the article/Supplementary Material, further inquiries can be directed to the corresponding author.
