# Supplementary material for: Case Report: Diverticulum of the left ventricular outflow tract: multimodal imaging
Source: Front Cardiovasc Med. 2024 Nov 20;11:1489688. doi: 10.3389/fcvm.2024.1489688 (PMC11614780; doi:10.3389/fcvm.2024.1489688)
Supplement: Supplementary file 1 [file Datasheet1.pdf]

SUPPLEMENTARY MATERIAL.

Supplementary Figures

Supplementary Figure S1： The digital subtraction angiography (DSA) results. RCA= right coronary artery, LCA=left coronary artery.

Supplementary Figure S2： The surgical results.

Supplementary Figure S3： The postoperative transthoracic echocardiography (TTE). AO =aorta, PA =pulmonary artery, LA= left atrium, RA= right atrium.

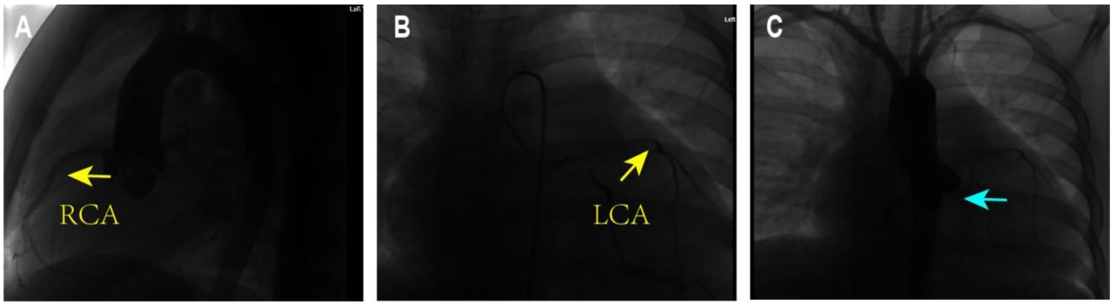

Figure S1. The digital subtraction angiography results (DSA).

The DSA results of the right (A) and left (B) coronary arteries showed no stenosis (yellow arrow). (C) DSA indicated the presence of the diverticulum (blue arrow). RCA= right coronary artery, LCA=left coronary artery.

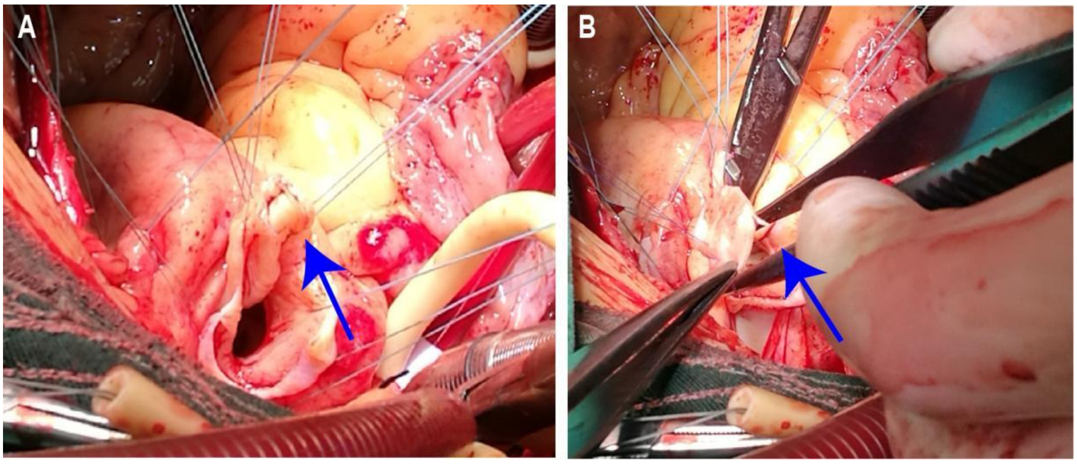

Figure S2. The surgical results.

(A) There was a rupture at the junction of the left and right pulmonary valve at the top of the capsule (blue arrow). (B) The 5 – 0 Prolene thread was used for the approximation of the pulmonary artery rupture (blue arrow).

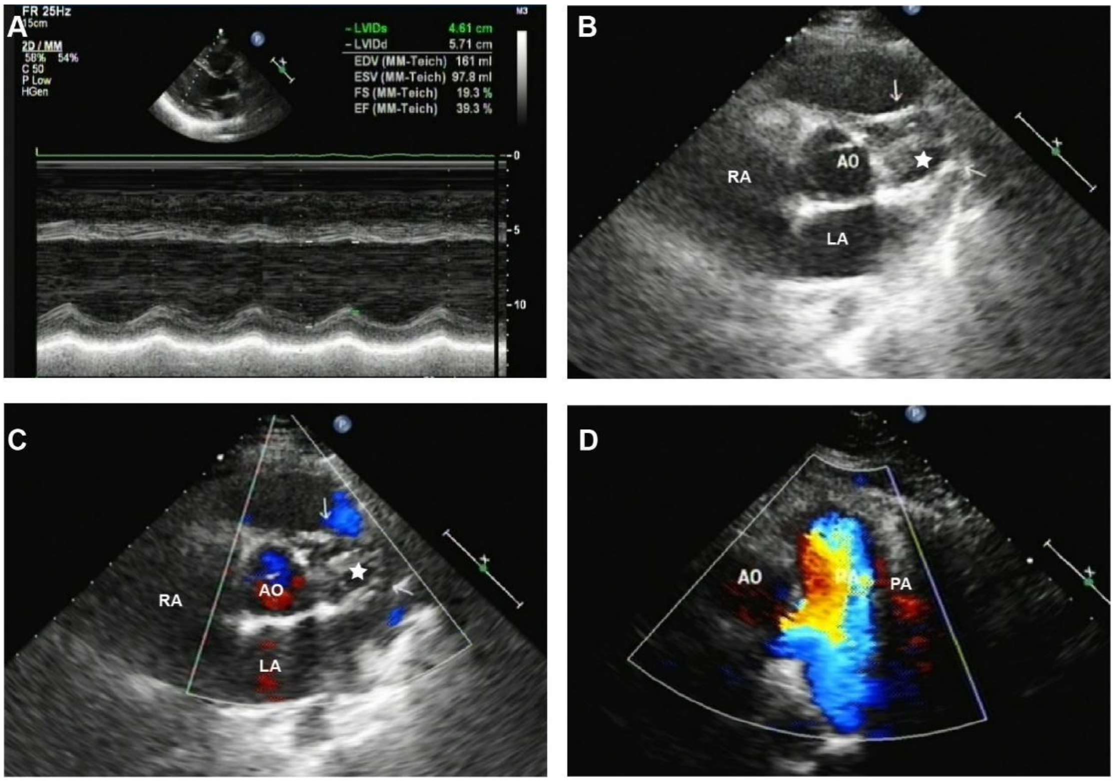

Figure S3. The postoperative transthoracic echocardiography (TTE).

(A) The postoperative echocardiography demonstrates that the left ventricle was still dilated. **(B and C)** The diverticulum collapsed without any residual shunts. **(D)** Unobstructed blood flow in pulmonary arteries. AO =aorta, PA =pulmonary artery, LA= left atrium, RA= right atrium.
